# Acceptability by community health workers in Senegal of combining community case management of malaria and seasonal malaria chemoprevention

**DOI:** 10.1186/1475-2875-12-467

**Published:** 2013-12-30

**Authors:** Roger CK Tine, Pascal Ndiaye, Cheikh T Ndour, Babacar Faye, Jean L Ndiaye, Khadime Sylla, Magatte Ndiaye, Badara Cisse, Doudou Sow, Pascal Magnussen, Ib C Bygbjerg, Oumar Gaye

**Affiliations:** 1 Service de Parasitologie, Faculté de Médecine et Pharmacie, Dakar, Sénégal; 2 Agence Européenne pour le Développement et la Santé, Bruxelles, Belgique; 3 Clinique des Maladies Infectieuses, Centre Hospitalier Universitaire de Fann, Dakar, Sénégal; 4 Department of International Health, Immunology and Microbiology, Faculty of Health Sciences, University of Copenhagen, CSS Øster Farimagsgade 5 Dk 1014, Copenhagen, Denmark

**Keywords:** Malaria, Chemoprevention, Acceptability, Children, Senegal

## Abstract

**Background:**

Community case management of malaria (CCMm) and seasonal malaria chemoprevention (SMC) are anti-malarial interventions that can lead to substantial reduction in malaria burden acting in synergy. However, little is known about the social acceptability of these interventions. A study was undertaken to assess whether combining the interventions would be an acceptable approach to malaria control for community health workers (CHWs).

**Methods:**

Sixty-one interviews and six focus group discussions were conducted nested in a cluster-randomized trial assessing the impact of combining CCMm and SMC in a rural area of Senegal. Participants consisted of: (i) members of village associations, (ii) members of families who had access to the interventions as well as members of families who did not access the interventions, (iii) CHWs, and (iv) community leaders, e g, religious guides and village chiefs.

**Results:**

The interventions were acceptable to the local population and perceived as good strategy to make health care services available to community members and thus, to reduce the delays in access to anti-malarial treatment as well as expenses related to patients’ transfer to the health post. The use of malaria rapid diagnostic test (RDT) contributed to improving CHWs diagnostic capacity as well as malaria treatment practices. Study participants notified RDT and drugs stock-out as the major risk for sustainability of the intervention at community level.

**Conclusion:**

Combining CCMm and SMC is a well accepted, community-based approach that can contribute to control malaria in areas where malaria transmission is seasonal.

## Background

Recently, the World Health Organization (WHO) advocated the scaling up of malaria control interventions in order to reduce malaria burden in areas where malaria is still a public health problem [1,2] and efforts are being made by sub-Saharan countries to reach that goal [3]. Community implication is central to controlling malaria. However, it requires a good level of acceptability of anti-malarial interventions by community members and community health care providers. It is recognized that community health workers can play a major role in promoting public health interventions and make valuable contributions to the performance of health systems [4,5] and delivery of primary health care [6,7]. In Senegal, the total number of health huts headed by community health workers (CHWs) is estimated at 1,703; these health huts represent the primary point of care in many rural areas [8].

Malaria control in some areas will require a strategy using a combination of anti-malarial interventions [9]. Prompt access to diagnostic testing and effective treatment with artemisinin combination therapy (ACT) is a fundamental element of malaria control. Community case management of malaria (CCMm), defined as the provision of anti-malarial treatment at household level [10], is one approach to improving access to prompt treatment with effective anti-malarial drugs, such as ACT, at community level. Seasonal malaria chemoprevention (SMC), previously referred to as intermittent preventive treatment in children (IPTc) [11] is a new strategy aiming at reducing malaria morbidity and mortality in children living in areas where malaria transmission is highly seasonal [11]. Studies conducted in sub-Saharan Africa demonstrated that adding SMC to CCMm can significantly reduce malaria incidence [9,12,13].

An intervention may have a high level of efficacy under clinical research conditions, but this does not necessarily mean that the communities for which it is intended will accept the intervention. This may reduce the effectiveness of the intervention. Faye *et al.*[14] demonstrated that, with the introduction of malaria rapid diagnostic tests (RDT) in primary health care units in Senegal, changing behaviour regarding diagnostic and treatment practices requires significant efforts and additional motivation from health care providers. Many sociocultural parameters can influence community acceptability of a new intervention [14] but a well accepted intervention can easily be disseminated throughout the community. Although the combination of SMC and CCMm has been shown to improve malaria control, little is known about its social acceptability [9,12,13]. This study was thus undertaken to assess whether combining CCMm and SMC is an acceptable malaria control approach both to CHWs and community members.

## Methods

### Study settings

The study was carried out at the Bonconto health post, located in the Velingara health district in the southeastern part of Senegal, 500 km from the capital city of Dakar. The health post is headed by a nurse and oversees eight health huts in eight villages staffed with CHWs, serving a total population of 10,016 inhabitants. These health huts represent the primary point of care of febrile patients. In this area, malaria transmission is seasonal, occurring during the rainy season (July to November) with a peak transmission in October and November. *Plasmodium falciparum* is the predominant parasite species, and transmission is mainly due to *Anopheles gambiae s.l.*[15]. In 2010, the National Malaria Control Programme (NMCP) initiated a strategy to achieve universal coverage with long-lasting, insecticide-treated nets (LLITN) in this area. By end 2010, the overall coverage of LLITN in the study area was 95.6% [12].

### Study design

Eight villages located around the Bonconto health post were selected for the implementation of an integrated malaria control strategy combining CCMm and SMC. To assess the acceptability of the combined intervention, a qualitative study was carried out in parallel to a cluster-randomized trial aiming to evaluate the impact of combined interventions on malaria burden as described elsewhere [12]. A household survey was conducted prior to the implementation of the intervention to assess community knowledge towards malaria as well as malaria health-seeking behaviour.

### Interventions implementation

The intervention has been described elsewhere [12]. In brief, eight CHWs in the eight villages around the Bonconto health post were trained to diagnose malaria using RDT and to provide prompt treatment with artemether-lumefantrine (AL) (Coartem®) to children < ten years. Children involved in the trial were assigned to a weekly follow-up visit and during each scheduled visit axillary temperature was measured for each child. Children who presented with fever without any obvious non-malarial cause of fever were tested with an RDT. Patients with uncomplicated malaria were treated by the CHW with AL. Children presenting with severe malaria received one artesunate suppository (10 mg/kg), prior to referral to the Bonconto health post. Both AL and malaria RDT (Malaria Ag SD Bioline™) were supplied by the NMCP. Four CHWs were randomly chosen to also administer monthly SMC, comprising a single dose of sulphadoxine-pyrimethamine (SP) plus three doses of amodiaquine (AQ) (Kina Pharm^LTD^) (taken daily for three days) on three occasions during the transmission season. SP and AQ dosing was according to age [12].

### Community health workers’ (CHWs) training and supervision

The CHWs involved in the implementation of the interventions were selected by community members. An initial training on the management of malaria cases was done by the NMCP. In routine practice, these CHWs were supervised monthly by the district staff. All CHWs had been involved in delivering primary health care services for at least one year before the start of the study.

Prior to the start of the study, another round of training focused on the identification of malaria cases using RDT, on the treatment of uncomplicated malaria cases with AL, the identification of severe malaria cases and the administration of pre-referral artesunate was done by the study team (medical doctors and laboratory technician working at the Department of Parasitology, University of Dakar). The NMCP guidelines were used to train CHWs. They were also trained on the study procedures. During the study period, weekly supervision was carried out by the local clinical monitor.

### Study conceptual framework

In the study framework, acceptability is defined as a population’s attitude, perception and adherence to intervention as well as barriers that can influence the usage of anti-malarial interventions. Based on these assumptions, the study examined the acceptability of combining CCMm and SMC, both by stakeholders and community health care providers. The framework recognized that the decision to use an intervention depends on several parameters among which: (i) population knowledge and information on the intervention, (ii) the potential effect of the intervention, and (iii) population’s perception, attitudes regarding the disease itself and existing control measures. The study’s working frame was that acceptability is a combination of positive perception, correct understanding and adequate usage. If acceptance is high it will lead to promotion and participation.

### Data collection methods

Focus group discussions (FGDs) and face-to-face, in-depth interviews (IDIs) were performed by trained researchers with backgrounds in clinical and social sciences; the researchers were required to be fluent in the local language (*Pular*). All FGDs and IDIs were recorded and researchers were advised to minimize as much as possible note intake, to allow a full interaction between interviewer and interviewee; notes were taken only for precision to complement audio records.

FGD and IDI guides were developed to capture the community’s perception and CHWs’ acceptance of the introduction of the interventions. The IDI guide included closed and open questions that focused on the study objectives. All data collection tools were pre-tested in Velingara City, which was not one of the study sites. The same researchers collected data during pre-testing and during the main study.

A total of 61 interviews and six FGDs were conducted. The participants were: (i) members of village associations, (ii) members of families who had access to the implemented interventions and those who did not have access, (iii) the caregivers (nurse, CHWs, matrons and CHWs’ assistants *‘relais’*), and (iv) community leaders such as religious guides, village chiefs, group leaders. The average duration and the number of participants per FGD ranged respectively from 30 minutes to one hour and four to eight participants. Table 1 summarizes study participants’ profiles.

**Table 1:** Study participants’ profile and distribution of qualitative methods

	**In-depth interviews**	**Focus group discussion**
**TOTAL**	**61**	**6**
**Groups and associations**	**6**	**4**
Village association		2
Health committee	4	
Women group		1
Other groups (Mbotay, Tontine*)	2	1
**Households**	**30**	
Beneficiaries of the intervention	15	
Non-beneficiaries of the intervention	15	
**Caregivers**	**16**	**2**
CHWs	8	1
CHWs’ assistants (Relais)	4	1
Matrons	4	
**Community leaders**	**10**	
Imam**	2	
Village chiefs	4	
Rural community authority	1	
Marabout***	2	

*A mbotay is a women friendship based group and a tontine is a saving mechanism organized by women (local banking).

**The Imam chairs the Muslims’ praying ceremonies.

***The Marabout is a Muslim religious guide who can provide religious education.

### Data analysis

For the quantitative survey, data were entered in Excel™ software and analysed using STATA IC 12. Continuous data were described in terms of mean and standard deviation and categorical data were described with percentage. IDIs and FGDs were recorded in *Pular*, the local language in the study area. Recordings were transcribed and then translated in French (with a revision proceeded twice). Interview transcripts were coded with QSR NVivo 8™ software; the coding was used to identify recurrent themes using a logico-semantic method and a thematic analysis was done from the information generated by IDIs and FGDs.

### Ethical consideration

The project was discussed with community members prior to any investigation and community consent was obtained from community leaders (religious guide, village head). The study protocol was approved by the National Ethics Committee of Senegal (Conseil National de Recherche en Santé). Approval N 027/MSP/DS/CNRS, 18/03/2010). In the field, written informed consent was obtained from all participants before participation in interviews and focus groups.

## Results

### Study community characteristics

During the baseline household survey, 225 mothers were interviewed using a standard questionnaire with close-ended questions. Participants showed correct knowledge of malaria, including association between malaria and mosquito bite (92%), fever as main symptom (94.4%); 52% of respondents would seek treatment within 24 hours of onset of malaria symptoms at the health hut, 34.6% at the health post, while 11.6% would self-medicate either with drugs available at home or with purchased drugs (mainly paracetamol), and 1.8% would primarily consult traditional healers. Mothers perceived bed nets as central to preventing malaria (93.8%) and bed net ownership was 99.1% (Table 2).

**Table 2:** Participants’ characteristics during the household survey conducted at baseline (N = 225)

**Parameters**	**Frequency**	**Percentage**
Age in years (mean SD)	27.6 ± 8.0	--
**Ethnic group**		
Peulh	218	96.9
Socé	03	1.3
Others (*Wolof, Sèrère, Bambara*)	03	1.3
**Instruction level**		
Illiterate	176	78.2
Primary school	37	16.4
Secondary school	07	3.1
Religious school	05	2.2
**Professional activities**		
Unemployed	189	84.0
Farmer	22	9.8
Trader	14	6.2
**Malaria health-seeking behaviour**		
Declared to seek treatment at health hut	117	52
Declared to seek treatment at health post	78	34.6
Self-medication	26	11.6
Declared to seek treatment to traditional healers	04	1.8
**Knowledge on malaria**		
Caused by mosquito bites	207	92
Fever as main symptom	213	94.7
**Cited malaria preventive measures**		
Bed nets	211	93.8
Indoor residual spraying	40	17.8
Environment sanitation	28	12.4
**Bed net ownership**	**223**	**99.1**

### Perception of the interventions

The local population considered CCMm and SMC as acceptable interventions to make health care services available for community members and thus to reduce delays in access to anti-malarial treatment as well as expense related to patients’ transfer to the health post. The economical advantages and improvement of access to health care were expressed as follows:

During the rainy season, access to the health post is very difficult and can take many hours. In addition, sometimes the required financial resources for the trip to the health post are not available. When people do not have to go to the post to seek treatment, it’s a great advantage.

FGD, Women group for respondent 2, R2

Community members considered that preventive measures such as SMC represent an important component of malaria control as this strategy can contribute to further reducing the occurrence of malaria among children. It is perceived also as a means to improve the quality of life of children.

It is much easier to prevent a disease than to treat sick individuals.

FGD, Pregnant women solidarity club, R3

A child who takes the drugs has less risk of developing malaria attacks than a child who did not take the drugs. Malaria can deeply affect a child and even after adequate treatment, it can take time before the child fully recovers. The use of drugs to prevent malaria for children has contributed to reducing the number of children who need to be transferred to the Health post for malaria treatment.

FGD, Pregnant women solidarity club, R5

CHWs appreciated the project because they believed it to be a good opportunity to strengthen quality of malaria diagnosis and to improve access to anti-malarial treatment. The project has contributed to increasing the level of confidence of the CHWs during their daily activities, as well as their level of consideration within the community.

The project was well received in each village. People are now seeking care at the health hut for any disease, including malaria. The level of trust in our work from the population is now increased and our relationship with the population has improved. This has been achieved because the project has contributed to reducing the number of malaria cases in the community. In addition, the use of RDT has been very helpful in having a correct diagnostic.

IDI, CHW

*There is an improvement with the new method as before there were problems of diagnosis. Some signs were similar and we could not differentiate them. Fortunately, with the RDT, we are informed immediately on malaria diagnosis.* FGD, CHW, R1

### Population’s adherence and satisfaction with the interventions

The adherence to the project was mainly motivated by the expected advantages and benefits. This has led to some changes in terms of health-seeking behaviour.

Habits have changed. In the past we used to go to the neighbouring village where there is a traditional healer or we give herbal to sick children. Treatment methods have changed completely.

IDI, a woman in village

Women discuss a lot about the doctor, I will not say they have stopped the traditional methods, but they are more likely to consult at the health hut.

IDI, a health community leader

We used to do self-medication with paracetamol. Tablets were bought from the shops and given to children. They will not necessarily cure but will look better. With the use of RDT, malaria cases are correctly diagnosed and treated immediately.

FGD, Women group for respondent, R1

There was little doubt or fear expressed by the stakeholders, and the adherence to the project was good, induced by the communication plan set in place prior to the project implementation, as well as the effect of the interventions on the population’s health. In addition, local authorities were sensitized and asked to collaborate on communication processes.

We have been sensitized by the CHW on the project procedures and the advantage of the use of malaria RDT as well as the use of drugs to prevent malaria.

IDI, Woman in Sare Bossedie village

At the beginning of the project, I had some doubts. After the first visit, I was reassured because the drug was safe. In addition, my child did not get malaria this year.

FGD, Mbotay, R1

I was afraid because my child was very ill. The CHW took some blood from my child’s finger; this leads me to believe that it was a very severe disease. Later, I understood that the blood was taken to check whether my child had malaria or not. After the results, the CHW gave my child some tablets. A few days later, my child was doing very well.

FGD, Mbotay, R4

I thought my child was going to die because he presented fever and convulsion. At the health hut, the CHW took blood to check for malaria. He said that my child was suffering from severe malaria. He administered to the child a suppository and asked me to take the child to the health post. Before our arrival to the health post, the convulsions had stopped and the fever had decreased.

IDI, woman in Diang Diang

However, despite this participatory approach, some leaders regret the change from traditional methods. A village chief gives the following position (with an air of regret):

In the past, people often consulted traditional healer who diagnosed a disease and treated it with decoctions.

[How did traditional healer carry out the diagnosis?]

By palpation, he then said that the patient was suffering from a particular disease. But today people are more likely to go to the health hut.

IDI, village chief

### Potential determinants of the promotion of the use of RDT, ACT and SMC

The promotion of the integrated malaria control strategy with community case management and SMC can be influenced by several factors, including availability of drugs and RDT. Although CHWs are very well motivated to promote the strategy, repeated stock-outs can negatively influence the use of this strategy. This was confirmed in CHWs’ and community members’ opinions when they were interviewed on the potential barriers that can influence the promotion of the strategy.

Today we can obtain drug at the health hut. But any time there are stock-outs for the drug, we have to go to the Bonconto health post, which is 12 km away from our village before we can get access to any treatment. This is time consuming and the travel conditions are so hard.

FGD, woman in Bantancountou

As long as you can provide anti-malarial treatment after a positive RDT, mothers will adhere to the strategy. In a situation of anti-malarial treatment stock-out, I can perform a rapid diagnostic test but cannot deliver treatment if it is positive; in such a situation, the mothers may lose faith in my work and may not adhere to the programme.

FGD, CHWs’ assistant, R3

The implication of community leaders has been recognized as an important factor that can ensure the promotion of the effective use of anti-malarial interventions, as suggested by CHW during focus group discussions.

The chief of the village, the religious guide and other village leaders have agreed on one principle: if I am aware of any parents who refuse to take their child to the health hut when the child is sick, I have to inform them. This is because they consider that in terms of malaria, anyone who refuses to use preventive methods or to be treated is a danger for the community.

FGD, CHW, R5

A certain level of solidarity between CHWs and their community members is needed to keep the intervention sustainable. Indeed, CHWs consider that they are committed to their communities, however this is done at the expense of their own domestic productivity. They expect to receive some advantages or a certain contribution from the community in their living conditions. A CHW highlighted this:

I am a farmer and during the rainy season while people are farming, I am alone in the health hut taking care of individuals who need treatment and there is nobody who can work for me in the field; in this way, I may not harvest. The villagers have chosen us to do this work so we must find a solution to our problem because we have to feed our families.

IDI, CHW 3

It was recognized that people’s level of trust and confidence in CHW treatment practices is a key element that can influence population adherence to the interventions. This is extremely dependent on CHWs’ supervision and training. Although treatment practices are an important factor, tolerance and safety of the interventions need to be taken into account.

We trust him because he is competent and his treatment is good and effective. However, some children have some difficulties swallowing tablets and they sometimes vomit the tablets.

IDI, matron, R3

## Discussion

This study investigated acceptability of combining CCMm and SMC in rural areas of Senegal. The use of malaria RDT, although requiring blood intake, was well appreciated and both community members and CHWs acknowledged RDT as an accurate means of improving malaria diagnosis. However, stock-outs of RDT and drugs (AL) represent a major fear by both CHWs and stakeholders. In the event of stock-outs of anti-malarial treatment, CHWs are able to refer patients to the health post were they can be treated, but this is very costly and access to the health post can be problematic under certain conditions. Indeed, in many situations the required resources to seek treatment at the health post are not available and communities are forced to sell some possessions, for instance, livestock. This situation has been described previously by some authors as ‘catastrophic health expenditure’ and the impact on household resources has been discussed [16-20]. Despite efforts exerted by the NMCP in Senegal to control malaria, there is evidence that without continuous support from the health system to make the required supplies available, community engagement for the promotion of anti-malarial interventions will become problematic.

In terms of malaria health-seeking behaviour, community members are more likely to seek care at the health hut than from traditional healers and the referral system put in place (from the health hut to the health post) seems to be well respected. Although there is a small chance that compliance with the normal referral system in these localities may have been induced by the study itself, the high level of frequentation of primary point of care in these areas is an important element that can contribute to improving access to anti-malarial interventions. CHWs can also play a major role in the promotion of these interventions. Regular supervision and monitoring is however required to ensure that CHWs’ treatment practices remain in line with existing guidelines [5,21-23]. Community members’ level of trust and confidence for CHW practices is a fundamental element that can influence their adherence to SMC and CCMm. Stakeholders usually refer to CHWs’ training and supervision as a means to reach a good level of confidence and trust.

The promotion of anti-malarial interventions can be influenced by the cultural context. In this study where the *peulh* (‘Fulani’) represented the majority of the ethnic groups, it was noted that community leaders are important authority figures that can influence individual or even community behaviour. In such a context, it is important that these leaders have access to the right information regarding any intervention so that they can convince community members to adhere to the use and promotion of these interventions. Thus, knowledge capacity strengthening activities should target both CHWs and community leaders who can contribute to the prevention of the disease. This can be achieved by a better understanding of the local and traditional means of communication [24].

The introduction of a new intervention can contribute to the reduction of the burden of the disease; moreover, it can have an impact on social relationships within the communities who benefit from the intervention. Indeed, the study has led to a better consideration of the role of CHWs within their respective communities and more credibility is now given to the CHW treatment practices compared to that given to traditional healers. This can however lead to conflict, particularly when traditional healers perceive the CHWs as a threat to their own credibility.

The study showed that community members adhered to the integrated malaria control intervention because of its efficacy in reducing malaria burden. The implication of CHWs in the promotion of anti-malarial interventions has contributed to maintaining a high level of coverage and usage of both CCMm and SMC at community level [12]. CHWs usually represent the first individual who can offer primary health care and they can help to better organize existing health systems [7,22,25,26]. They are community members and they are committed to their own communities [27]; they can thus help to reach individuals who live in remote areas where access to health care is problematic [28]. However, CHWs’ place in health systems is still in debate [29]. Despite an important level of commitment by CHWs to promote access to anti-malarial interventions in their own communities, economical reasons can affect the sustainability of community-based interventions. Without appropriate systems of compensation, retention of CHWs will be difficult to ensure [30].

### Study limitations

The main trial was aiming at assessing the impact on malaria of combining CCMm and SMC. As a line extension of the cluster randomized trial, this qualitative study could have followed a comparative design to compare attitudes and acceptability between communities. However, despite this limitation, the study has generated evidence that CCMm and SMC are well-accepted interventions by rural communities.

## Conclusion

Combining CCMm and SMC is a well-accepted, community-based approach that can contribute to malaria control in areas where malaria transmission is seasonal. CHWs can play a major role in the promotion of SMC and CCMm, but regular supervision and monitoring will be required to ensure that CHW treatment practices remain in line with standard quality of care. Community leaders’ engagement in the promotion of anti-malarial interventions can contribute to improving the efficiency of community-based interventions.

## Competing interests

The authors declare that they have no competing interests.

## Authors’ contributions

RCKT, PN, CTN, PM, ICB, and OG conceived and designed the study. PN was responsible for the conception of the interview and focus group guides. RCKT and PN trained the field workers and supervised the data collection. PN performed all the qualitative analysis. RCKT wrote the first draft of the manuscript. All authors read and approved the final manuscript.
